# Tracing dividing stem cells

**DOI:** 10.18632/aging.101491

**Published:** 2018-07-01

**Authors:** Oleg Podgorny, Natalia Peunova, Grigori Enikolopov

**Affiliations:** 1 Center for Developmental Genetics and Department of Anesthesiology, Stony Brook University, Stony Brook, NY 11794, USA; 2 Koltzov Institute for Developmental Biology, Moscow 119334, Russian Federation

**Keywords:** stem cell division, triple S phase labeling, quadruple labeling, combinatorial labeling

To answer crucial questions in cell biology, neuroscience and cancer research, we must be able to track dividing cells and determine the parameters of the cell cycle. This task is particularly challenging when it comes to stem cells, which undergo a cascade of transitions to arrive at various fates: a given cell might remain quiescent, or emerge ready to initiate the division cycle, re-enter the cycle, return to quiescence, get eliminated, or differentiate. These transitions thus create a complex range of stem cell subsets that differ by cell cycle stage, number of preceding duplication cycles, propensity to return to quiescence, and extent of differentiation. These subsets likely play different roles in development and tissue repair, and respond differently to therapies – a complexity particularly pertinent in the developing and adult brain, where stem cells give rise to new neurons, and in tumors, where stem-like cells underlie neoplastic growth.

To assess stem cell division and fate, researchers label dividing subsets and trace their lineage, most informatively by using nucleotide analogs to tag distinct cohorts of cells engaged in DNA replication, and then revealing and phenotyping those cohorts (a procedure often called “birthdating”) [1]. Most often, cells traversing the S-phase are tagged with halogenated nucleotides (e.g., bromo-, chloro-, or iodo 2’-deoxyuridine, BrdU, CldU, and IdU) which can be detected using specific antibodies; with terminal alkyl-bearing nucleotides (e.g., 5-ethynili-2’-deoxyuridine, EdU), revealed through cycloaddition (click-chemistry); or with radioactive nucleotides (e.g., tritiated thymidine), revealed using radiography. This approach can be enhanced by following endogenous markers of cell division (e.g., PCNA or Ki67 proteins) and lineage markers (e.g., those provided by GFP-expressing reporter transgenic lines). Furthermore, these DNA tags can be combined to detect defined cell cohorts; such double S-phase labeling approaches have been ingeniously used to follow stem cell fate in the developing and adult nervous system [2,3].

Clearly, ability to distinguish between three (or more) DNA tags in parallel would exponentially increase the power of the labeling approach, revealing two distinct cell cohorts for one DNA label (i.e., labeled and non-labeled), eight for two labels, over a hundred for three labels, and over thirty thousand for four labels (Fig. 1).

**Figure 1:**
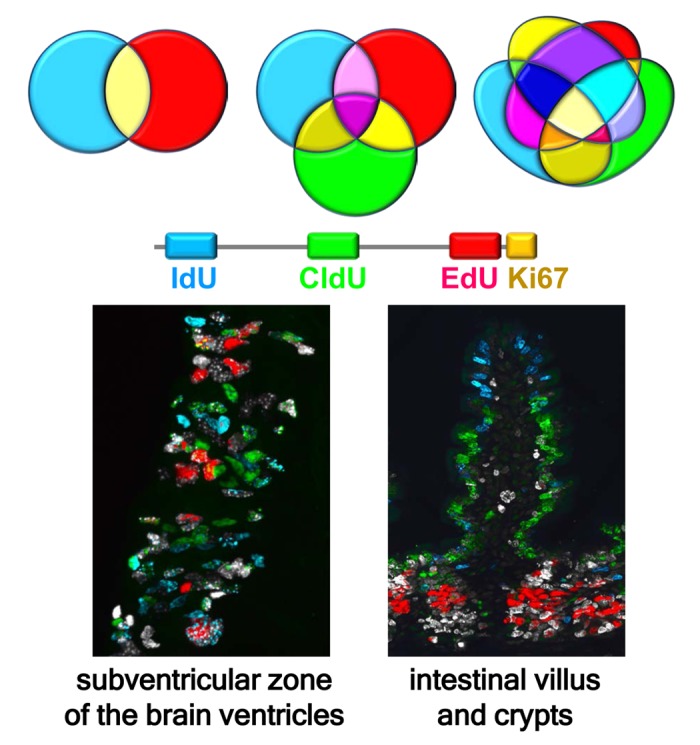
**Multiple labeling of dividing cells reveals defined subsets of stem and progenitor cells and their progeny.** The number of differentially labeled subpopulations of cells expands exponentially as the number of individual tags and their possible combinations increases, even if only a minority of them correspond to biologically meaningful cell cohorts. Shown is an example of quadruple labeling of dividing cell cohorts in the subventricular zone (SVZ) of the lateral ventricles (left) and in the villi and crypts of the intestine (right). IdU, CldU, and EdU were sequentially injected in mice every 24 hr and the tissues analyzed 2 hr after the last injection, revealing three injected DNA tags and Ki67, an endogenous marker of dividing cells. Cells labeled with one, two, three, and four markers can be observed. Labeling of the brain shows currently or recently dividing neural stem and early progenitor cells and their progeny in the SVZ. Labeling of the intestine reveals rapid migration of newborn cells from the crypts to the top of the villi, with blue IdU-positive cells at the top of the villus, green CldU-positive cells in the middle segment of the villus, red EdU-positive cells strictly in the crypts, and white Ki67-positive cells in the crypts and the basal segment of the villus. Zones of non-overlapping colors reflect rapid ascent of enterocytes, born in the crypts, along the villi.

Even if most of those cohorts do not carry biologically relevant information, some do correspond to important subsets that cannot be otherwise revealed. Still, pushing beyond two labels has been a remarkably stubborn problem, as cross-reactions between the antibodies and non-cognate tags typically preclude a clear separation of the cell subsets or lead to artifacts.

Recently, however, we have developed a robust new technique for triple labeling dividing cells, with a fourth label allowing us to phenotype stem cells and their progeny, or otherwise identify dividing cells in more granular detail [4]. Specifically, we are able to simultaneously detect three S phase tags (CldU, IdU, and EdU), supplementing them with an additional cell cycle marker such as Ki67, which provides the fourth label for cell division, or by a cell fate reporter such as Nestin-GFP transgene, which allows us to phenotype the dividing cell cohorts. When we applied our method to various tissues, we imaged the patterns of cell division in never-before-seen resolution, highlighting a conveyer-belt-like pattern of division and migration of newborn enterocytes along the intestinal villi and division of stem cells in the crypts [5]; distribution of spermatocytes at different stages in the seminiferous tubules; and distinct cohorts of neural stem and progenitor cells in the neurogenic regions of the adult brain (the subventricular zone and dentate gyrus) [6] (Fig. 1).

Our triple S-phase labeling method has broad potential, particularly in revealing a vast diversity of subsets of stem cells and their progeny. To illustrate, using combinatorial scoring, we can identify various functionally important cohorts: from cells that have made their first entry into the division cycle after a defined period of time, to cells that have re-entered the division cycle; to cells that have been dividing, then entered quiescence and then started dividing again; to cells that have been quiescent, then divided, and then re-entered quiescence; to cells that have survived at a particular stage of the programmed elimination process; or to cells that have been continuously dividing. In the majority of cases, these subpopulations of cells cannot be identified using the conventional single- or-double-S-phase labeling approaches.

Besides birthdating, this technique can also use it to modify and combine cell cycle label paradigms (e.g., applying long-term cumulative labeling vs. a pulse injection) and create new experimental designs (e.g., combining DNA labeling with lineage tracing, drug testing, or both). Also note that cell subpopulations are revealed in the same tissue in parallel, thus decreasing the scale of experiments and increasing their reproducibility.

To further improve on our method, we envision several strategies, for instance, increasing the number of tags and honing the methods of their detection; defining the range of tags’ doses that allow robust detection with minimal effect on various cell processes and metabolism; automating 3D detection and analysis of the cell cohorts [7]; adjusting the technique to the expanding variety of methods for tissue clearing; and combining the technique with advanced cell cycle-specific reporters and genetic lineage tracing approaches.

## References

[1] Kuhn HG, et al. Cold Spring Harb Perspect Biol. 2016; 8:a025981. 10.1101/cshperspect.a025981 26931327 PMC4772100

[2] Vega CJ, Peterson DA. Nat Methods. 2005; 2:167–69. 10.1038/nmeth741 15782184

[3] Takahashi T, et al. Proc Natl Acad Sci USA. 1994; 91:375–79. 10.1073/pnas.91.1.375 8278397 PMC42950

[4] Podgorny O, et al. Stem Cell Reports. 2018; 10:615–26. 10.1016/j.stemcr.2017.12.020 29358087 PMC5830949

[5] Clevers H. Cell. 2013; 154:274–84. 10.1016/j.cell.2013.07.004 23870119

[6] Gonçalves JT et al. Cell. 2016; 167:897–914. 10.1016/j.cell.2016.10.021 27814520

[7] Shuvaev SA, et al. Front Neuroanat. 2017; 11:117. 10.3389/fnana.2017.00117 29311849 PMC5732941

